# Basal cell carcinoma- a rare clinical image

**DOI:** 10.11604/pamj.2021.40.145.30376

**Published:** 2021-11-09

**Authors:** Saritha Aadhi, Rakesh Krishna Kovela

**Affiliations:** 1 Sansa Gastro Liver and Skin Hospital, Patel Marg Bhuktapur Adilabad, Telangana, India,; 2 Department of Neuro Physiotherapy, Ravi Nair Physiotherapy College, Datta Meghe Institute of Medical Sciences, Sawangi, Meghe, Wardha, Maharashtra, India

**Keywords:** Basal cell carcinoma, hyperpigmented plaque, cancer

## Image in medicine

We are presenting a case of a 50-year-old female, a farmer by profession and often exposed to sunlight. She came with complaints of lesions on her face along with pain over the lesions for the past year, which was left unmanaged since then. No one in their family has similar complaints as per her knowledge. On thorough inspection of lesions, there was neither sensory loss nor oozing of any fluid. On evaluation, there were lesions over the nose and medial aspects of eyes looked like a hemorrhagic crusted plaque with hyperpigmented border initially started as hyperpigmented papules which progressed to present size gradually in one year. Skin biopsy was sent for histopathological examination which confirmed it as basal cell carcinoma, on the confirmation the patient was referred to the oncology department for further management.

**Figure 1:**
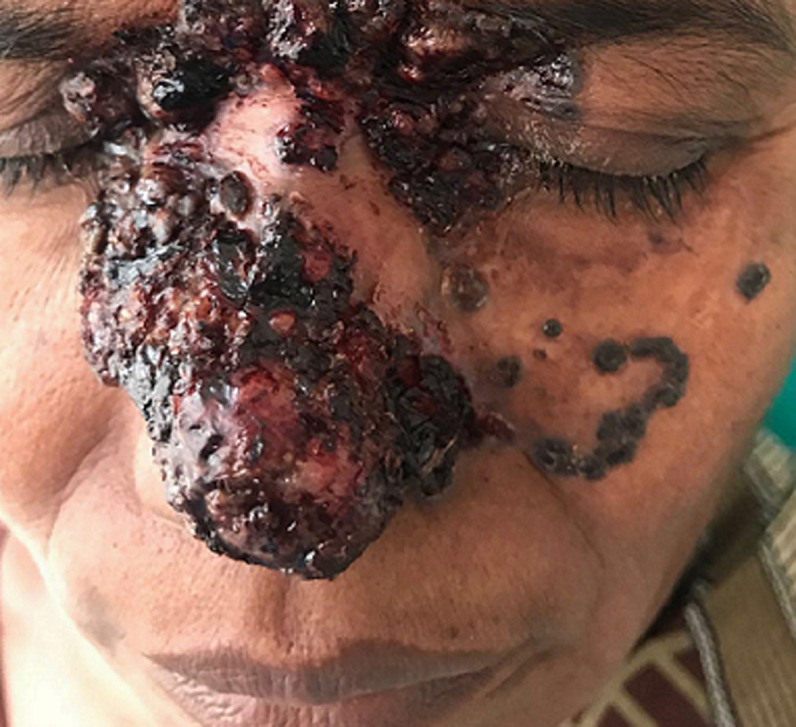
basal cell carcinoma

